# Nanoenabled Photothermal Materials for Clean Water Production

**DOI:** 10.1002/gch2.202000055

**Published:** 2020-10-14

**Authors:** Muhammad Sultan Irshad, Naila Arshad, Xianbao Wang

**Affiliations:** ^1^ Ministry‐of‐Education Key Laboratory for the Green Preparation and Application of Functional Materials Hubei Key Laboratory of Polymer Materials School of Materials Science and Engineering Hubei University Wuhan 430062 P. R. China; ^2^ Institute of Quantum Optics and Quantum Information School of Science Xi'an Jiaotong University (XJTU) Xi'an 710049 P. R. China

**Keywords:** clean water, nanoenabled, photothermal, steam generation

## Abstract

Solar‐powered water evaporation is a primitive technology but interest has revived in the last five years due to the use of nanoenabled photothermal absorbers. The cutting‐edge nanoenabled photothermal materials can exploit a full spectrum of solar radiation with exceptionally high photothermal conversion efficiency. Additionally, photothermal design through heat management and the hierarchy of smooth water‐flow channels have evolved in parallel. Indeed, the integration of all desirable functions into one photothermal layer remains an essential challenge for an effective yield of clean water in remote‐sensing areas. Some nanoenabled photothermal prototypes equipped with unprecedented water evaporation rates have been reported recently for clean water production. Many barriers and difficulties remain, despite the latest scientific and practical implementation developments. This Review seeks to inspire nanoenvironmental research communities to drive onward toward real‐time solar‐driven clean water production.

## Graphical abstract

Nanoenabled photothermal materials and their respective photothermal mechanisms, surface plasmon resonance, non‐radiative relaxation, and lattice vibrations are briefly described. The entire solar‐driven steam generation process, liquid to vapor generation, and vapor condensation along with a theoretical understanding of evaporation rate limitations is discussed. The bifunctional water‐energy nexus is reviewed for simultaneous water and electricity generation. In addition, the decisive challenges of this field are put forward.

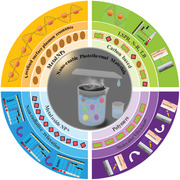

## 1 Introduction

**Figure 1:**
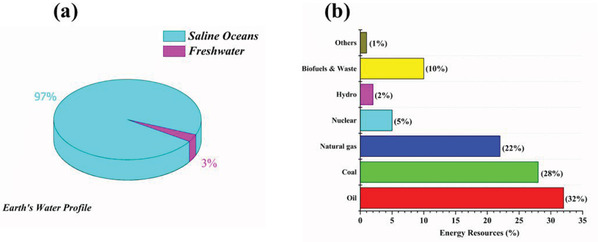
a) Current profile of water availability. b) Production of energy from different resources.^[^ ^5^, ^12^ ^]^

Water and energy are essential components of life for economic development and social change. The world's sustainability capacity will be severely stressed by clean water and energy resources that are intimately connected in the next decades.^[^ ^1^, ^2^ ^]^ Current technologies that counter water scarcity and energy problems at the cost of deteriorating environmental issues to improve access to water and energy cannot be solved on a sustainable basis.^[^ ^3^, ^4^, ^5^, ^6^ ^]^ Consequently, green technology has made tremendous efforts to generate clean water and electricity. Due to the increasing world population and the excessive use of limited water and energy sources, environment‐friendly renewable resources are required to meet this challenge.^[^ ^7^, ^8^, ^9^, ^10^, ^11^ ^]^ According to the current scenario, water‐energy availability nexus shown in **Figure** **1**.

Solar‐driven water evaporation is an auspicious technique to harvest maximum sunlight and offering solutions that reduce the environmental effect of freshwater scarcity and energy crisis.^[^ ^13^, ^14^ ^]^ However, its practical application is hampered by a low photothermal conversion efficiency of the volumetric system, which is limited by low absorption of the solar spectrum and heat loss.^[^ ^15^, ^16^, ^17^ ^]^ The recent invention of nanostructured solar absorbers has considerably drawn attention from the basic science of the solar‐powered evaporation of water, despite the growing importance of environmental protection and energy conservation.^[^ ^13^, ^16^, ^18^, ^19^, ^20^, ^21^, ^22^, ^23^, ^24^, ^25^, ^26^, ^27^ ^]^ The logical design of both nanomaterials significantly increased the efficiency of evaporation, using the interfacial heating principle, as efficient solar absorbers for solar‐driven evaporating systems. A solar‐absorbing interfacial heating system on the water surface reduces thermal losses in the water at the water‐air interface.^[^ ^17^, ^28^ ^]^

The sunlight is absorbed and transformed into heat by photothermal materials in a traditional solar‐powered evaporation system and utilized to create water vapors that condensed to harvest freshwater. Photothermal materials can efficiently absorb and convert light energy into heat. The produced heat could be served in various applications, including phototherapy, photoimaging, and clean water production.^[^ ^16^, ^19^, ^28^ ^]^ Over the last five years, there is a dramatically increased awareness about dealing with photothermal materials for solar water evaporation. The evaluation of zero CO_2_ emission of solar distillation to desalination from seawater is desirable and essential at a period in which global climate change, renewable energy, and water shortage are increasing, and growing.^[^ ^29^, ^30^, ^31^, ^32^ ^]^

With the emergence of many new materials for nanoenabled functional materials, including plasmonic metallic nanoparticles (NPs), graphene oxide (GO) and reduced graphene oxide (rGO), significant advances were achieved towards design and manufacture of innovative photothermal absorbers for solar‐powered water evaporation.^[^ ^4^, ^16^, ^19^ ^]^ The recently nano‐developed photothermal absorbers have reinvented the ancient solar distillation practice, becoming a modern green innovation for processing safe drinking water.^[^ ^28^ ^]^ This comprehensive review climaxes the exciting developments, particularly in the last five years, in terms of optimization, selection, and photothermal structural design along with their facing challenges. These challenges suppress the practical application of a solar‐powered desalination system, including salt accumulation, heat management, long term stability. Eventually, a premise and a possibility of a photothermal evaporation system will be predicted on behalf of theoretical investigations of salt accumulation, heat management simulation, excellent reported structural design. This analysis aims to enhance our understanding of the underlying phenomena behind the photo‐thermal conversion process in various nano‐absorber and provide guidance for the development of solar‐powered water evaporation and its future application, along with other fields such as energy and the climate.

## 2 Nanoenabled Photothermal Materials

In recent decades, the development of photothermal material has been very involved in water desalination to solve water deficit challenges. A good photothermal material should be proficient in harvesting the full range of the solar spectrum (200–2500 nm) intended for an efficient solar‐driven water evaporation system. The following problems are come upon while developing a suitable material for this purpose. First, the refractive index of inorganic photothermal materials should be more than two, resulting in a high reflection of light (>11%) by the Fresnel equation. Second, a flat surface's light absorption has an extreme response to the incident angle of the light. Such issues and problems are to be overcome entirely when thermoelectric materials utilized at the nano stage.^[^ ^13^, ^17^, ^28^, ^33^, ^34^ ^]^

**Figure 2:**
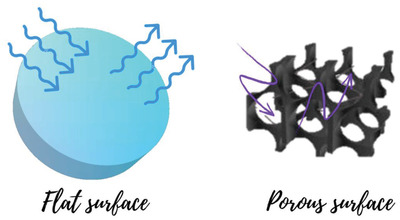
Comparative behavior of incident light on a flat and porous surface in terms of maximum light‐capturing within the matrix. Adapted with permission.^[^ ^28^ ^]^ Copyright 2018, The Royal Society of Chemistry.

In recent decades, fluids that fitted with nanoparticles (NPs) have been used in thermal absorbers to lessen surface heat loss, maintaining an even fluid temperature and increasing the thermal conductivity of fluids.^[^ ^13^, ^27^, ^35^, ^36^ ^]^ Owing to their surface plasmon resonance properties, NPs enabled them to absorb most part of solar spectrum along with conversion into heat energy when they subjected to sunlight. Thermal energy is transmitted rapidly from the NPs to water by direct contact with water and steam generation occurs due to the plasmonic effect of the NPs surface.^[^ ^27^, ^35^ ^]^ The highly porous structures can microscopically produce several reflections inside the matrix, which may lead to excellent light absorption, as illustrated in **Figure** **2**.^[^ ^28^, ^34^ ^]^ The described literature confirmed that the light absorption ability of the porous surface is superior to the flat surface, as recently reported cauliflower‐patterned Cu nanostructure, graphene foam, and metal nanoparticles modified wood.^[^ ^34^ ^]^

Nevertheless, thermal loss of heat from bulk water leads to a reduction in the efficiency of photothermal conversion. In order to overcome this problem, photothermal interfacial system integration improves converting efficiency and accumulates the heat within the photothermal matrix and avoid heat losses to bulk water.^[^ ^13^, ^15^, ^17^, ^28^ ^]^ The prescribed equation relates the photothermal conversion efficiency from sunlight to heat of nano‐absorber; η = *h*_LV_/*q*_i_ *C*_opt_, where η denotes the photothermal conversion efficiency and symbolizes the evaporation rates under sunlight, *h*_LV_ corresponds to enthalpy change of liquid‐vapor as well as sensible heat. *q*_i_ represents the solar intensity under 1 kW m^−2,^ while *C*_opt_ corresponds to multiple intensities of applied solar energy.^[^ ^13^, ^19^, ^34^, ^37^ ^]^ Finally, the plasmonic effects of plasmonic metals have specific wavelengths and are highly dependent upon shapes and sizes.^[^ ^13^, ^35^ ^]^ Metal nanoparticles with a large scale or different ways are combined in certain symmetry to obtain a broad range of solar power. Their light absorption capacity is the primary and foremost significant criterion for excellent photothermal materials.^[^ ^35^ ^]^

Although substantial efforts have been employed to achieve the highest light to heat conversion efficacy, it acquires sustainability for long‐term and less regarded.^[^ ^13^, ^14^, ^15^, ^20^ ^]^ In this virtue, numerous nanomaterials as a nanoenabled photothermal material over the past five years, such as carbon‐based materials, precious metals, metal oxides, and polymers, were intensively investigated with a high absorption level in the broad spectrum of solar energy. The sunlight, absorbed in a photothermal material, gives rise to a power field that energies mobile carriers within the material's crystals lattice and the energy obtained from the carriers becomes heat. In this context, we shall briefly present the latest progress of photothermal conversion phenomena in different materials such as carbon‐based nanocomposites, polymers, precious metals, and their oxides.

### 2.1 Nanoenabled Carbon‐Based Absorbers

Carbon enabled nano‐absorber to possess unique features of tightly held energy levels along with loosely π electrons that allowed to absorb the entire solar spectrum of sunlight. These π electrons absorbed sunlight and shifted to π* transition level, and then relax to their ground state via releasing energy in the form of heat.^[^ ^4^, ^28^, ^38^ ^]^ Several classes of carbon‐based absorber such as graphene oxide (GO), carbon black, graphite, carbon composites, carbon nanotubes (CNTs), and amorphous carbons exhibit outstanding solar to heat conversion capability owing to their vital inherent characteristics instead of metallic plasmon.^[^ ^37^, ^39^, ^40^, ^41^, ^42^ ^]^ The exceptional photothermal ability, minimum reflection and emittance, and excellent absorbance enabled carbon‐based materials as an efficient vapor generator. Moreover, the availability and cost‐effectiveness signify the importance of carbon‐based nano‐absorber, and they can endure acid, salty, and alkali environments.^[^ ^13^, ^24^, ^26^, ^28^, ^33^, ^38^, ^43^, ^44^, ^45^ ^]^ As compared to polymers, carbon can mold into various structures/designs equipped at macro to nano‐scale desired morphologies without any deterioration and exhibits long‐term efficacy, no environmental hazard that is required clean water production application. Due to the tunable light density of carbon‐based materials, it enabled them to float freely on the surface of the water that may lead to the insulating ability to minimize heat loss. Mostly, hydrophobicity prevails the floating capacity of the evaporator that is essential for a solar‐powered evaporation system.^[^ ^13^, ^26^, ^28^, ^33^, ^38^, ^45^ ^]^

**Figure 3:**
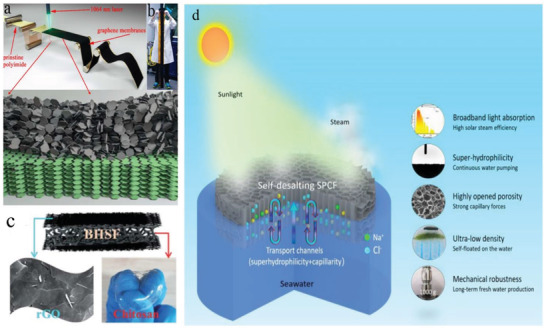
Carbon‐based nanoenabled absorber. a–b) Schematic illustration of the synthesis of laser‐induced graphene film a) graphical representation of laser‐induced Janus membrane on polyimide substrate along with structure of the Janus membrane deposition on a polyimide substrate b) the final optical image of human height laser‐induced GO thin film prototype. Reproduced with permission.^[^ ^46^ ^]^ Copyright 2018, The Royal Society of Chemistry. c) hollow spacer fabric (BHSF) based solar steam generation system manufactured via chitosan‐based filled spaces and surface modified by reduced graphene oxide. Reproduced with permission.^[^ ^47^ ^]^ Copyright 2019, The Royal Society of Chemistry. d) The schematic demonstration of a self‐desalted steam generator device equipped with superior characteristics. Reproduced with permission.^[^ ^48^ ^]^ Copyright 2020, The Royal Society of Chemistry.

Li et al. (2018), reported the Janus superhydrophobic/super hydrophilic porous graphene membranes with self‐righting, flexible, and high‐scalable features were fabricated via laser writing. These membranes provide benefits in applications for the solar steam generation with high efficiencies for practical use under one‐sun illumination along with cheap production, as demonstrated in **Figure** **3**a,b.^[^ ^46^ ^]^ Wang, Fei et al. (2019) also reported blank hollow spacer fabric (BHSF) based solar steam generation system manufactured via chitosan‐based filled spaces and surface modified by reduced graphene oxide resulting in excellent thermal insulation and mechanical stability which is measured up to 733 kPa compression strength at 70% strain and low thermal conductivity (0.08 W m^−1^ K^−1^). Solar evaporation efficiency was measured which was up to 86% under one sun intensity or 1 kW m^−2^, this solar generator gives an evaporation efficiency of 86%, along with efficient salt resisting properties, as demonstrated in Figure 3c.^[^ ^47^ ^]^

Yang et al. (2018), reported the fabrication of 3D cross‐linked honeycomb graphene foam material, which claimed to act as an ideal solar thermal converter enable of capturing and converting sunlight into heat and produce purified water under ambient conditions and low solar flux with very high efficiency. High specific water production rate of 2.6 kg m^2^ h^−1^ was achieved with near ≈87% photothermal conversion efficiency under one sun intensity and >80% efficiency even under ambient sunlight.^[^ ^49^ ^]^ Chen, Lihua et al. (2020) reported an approach for the fabrication of porous carbon nanofoam that was prepared by the carbonization of the pitch using the combination of CaCO_3_ and NaCl templates (PCN). The prepared PCN resulted in light‐to‐heat conversion efficiencies of 88%, 86%, and 84% under one sun, two sun, and three sun irradiations, respectively, which prove it as better thermal insulation, desired porous structure, and excellent light absorption.^[^ ^50^ ^]^ Qiu, Pengxiang, et al. (2019) stated the preparation of cost adequate and highly stable porous 3D carbon foams (CFs) prepared from natural wood by activating it with alkali which occurred having typically connected channels and rough surface. It enabled us to harvest 97% of the entire solar spectrum. The steam generation efficiency reported was 80.1% under 1 kW m^−2^ with excellent mechanical stability and high activation for seawater desalination.^[^ ^51^ ^]^ Wang, Chengbing et al. (2020) reported a self‐floating super hydrophilicity porous carbon foam (SPCF) for desalination system that acts as an integrated solar absorber fabricated from tomatoes (Figure 3d). 3D porous integrated structure (SPCF) enabled quickly dissolving salt from bulk water resulting in no apparent salt accumulation on the SPCF surface during continuous 8 h seawater desalination. Light absorption efficiency for SPCF was calculated up to (96.19%) with superfast solar‐thermal conversion capability (up to 92.7 °C temperature elevation under 2 sun illumination within 10 s), excellent mechanical robustness and reduced thermal conductivity, with an energy efficiency of 86% under one sun illumination equipped with a simultaneous response of salt resisting during vapor generation were obtained.^[^ ^48^ ^]^ Jang, Gyoung Gag et al. (2019), reported a scalable direct solar thermal carbon distillation (DS‐CD) tubular devices, manufactured via microporous graphite foam coated with carbon nanoparticle. DS‐CD based superhydrophobic materials that demonstrated effective solar absorption of over 96% and 64% solar–thermal conversion efficiency, respectively. The efficient solar absorption, controllable heat management, and porous nature of this structure enabled estimated direct‐solar desalination performance of ≈ 8.8 kg m^−2^ h^−1^ of permeate flux and ≈ 99.5% salt rejection under simulated concentrated solar–thermal irradiation.^[^ ^52^ ^]^

### 2.2 Nanoenabled Precious Metal‐Based Absorbers

Metallic nanoparticles have been thoroughly examined owing to their localized surface plasmon as an effective photothermal absorber in recent years.^[^ ^28^, ^35^, ^53^ ^]^ As the incident light frequency approaches the oscillation frequency of delocalized metal electrons, it triggers a collective excitation of the electrons, generating hot electrons. The hot electrons oscillate coherently with the incident electromagnetic field, resulting in heat generation by a joule mechanism. Surface plasmon induced localized heat to nanoparticle will be exchanged with the surrounding. Upon the illumination of fluid by sunlight, instant steam production takes place caused by the localized surface plasmon heating effect at the particle−liquid interface, resulting in encapsulation of metallic nanoparticles via a thin vapor bubble layer causing the reduction of thermal conductivity to some extent.^[^ ^53^, ^54^ ^]^ Studies show that an effective heat generation could be incorporated via crystalline nanoparticles comprised of several materials (such as Au, Ag, and semiconductors) in the presence of electromagnetic radiation.^[^ ^13^, ^14^, ^17^, ^28^, ^53^ ^]^

**Figure 4:**
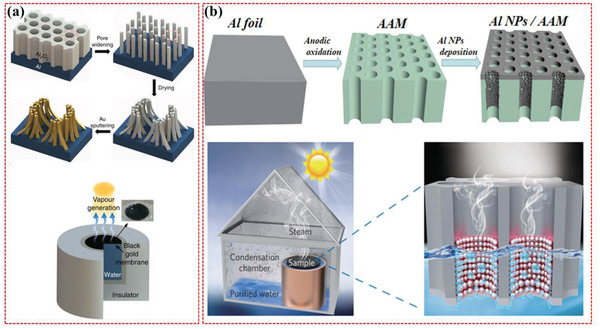
a) The overall schematic illustration of the fabrication of black gold photothermal membrane that is induced in hexagonal AAO pored matrix using pore‐widening procedure along with steam generator prototype structure, and the inset shows that black gold membrane floating on the water surface. Reproduced with permission.^[^ ^55^ ^]^ Copyright 2015, Springer Nature. b) the facile step by step synthesis and fabrication of plasmonic Aluminum nanoparticles (Al NPs) photothermal layer in which Al foils was utilized as a precursor for entire anodic oxidation process for AAM, and the desired Al NP/AAM structural design was achieved via Al NPs deposition on AAM, the schematic illustration of plasmon‐based steam generator prototype also presented. Reproduced with permission.^[^ ^56^ ^]^ Copyright 2016, Springer Nature.

Bae, Kyuyoung, et al. also reported the fabrication of flexible thin‐film black gold membranes with metallic nanoscale gaps (0–200 nm), resulting in average absorption of 91% at 400–2500 nm and the microscale funnel structures lead to ordinary reflection of 7% at 2.5–17 mm as illustrated in **Figure** **4**a.^[^ ^55^ ^]^ Recently, Zhou, Lin et al. reported a plasmon‐enhanced solar desalination device, fabricated by the self‐assembly of aluminum nanoparticles into a 3D porous membrane that could be float naturally on the water surface. The absorbing efficiency from the entire solar spectrum was >96%. The durability of the device showed a stable performance over 25 cycles under various illumination conditions, as demonstrated in Figure 4b.^[^ ^56^ ^]^ In this contribution, Zhou, Lin, et al. group reported a spectrum of selective plasmonic absorber using gold nanoparticles with an absorbing efficiency > 90% fabricated by a template‐assisted physical vapor deposition (PVD) process.^[^ ^57^ ^]^

Recent and vigorous work regarding metal nanoparticles (MNPs) was reported (Zhu, Mengwei, et al. (2017) in which a uniform distribution of fabricated MNPs based plasmonic material into the 3D mesoporous matrix of natural wood (plasmonic wood). Strong light absorption ability (≈99%) has been unveiled by plasmonic wood covering a wide wavelength band from 200 to 2500 nm, mainly caused by the plasmonic effect of metal nanoparticles and the waveguide effect of microchannels in the wood matrix. Due to the 3D mesoporous wood microchannels and nanochannels with less tortuosity can efficiently transfer water from the bottom to mainly referred to the capillary effect. Ultimately, 3D aligned porous structural design can accomplish a high solar conversion efficiency of 85% under ten‐sun illumination (10 kW m^−2^).^[^ ^58^ ^]^

Other plasmonic metal nanomaterials have also been investigated towards solar evaporation for clean water production. Liu, Changxu, et al. reported a reusable biomimetic super‐dark met the surface of 200 nm thickness that attains a solar thermal efficiency of 87% when exposed to an intensity of only 2.3 suns, maintaining a stable efficiency of 90% at higher solar intensities. The met surface is mainly comprised of enormously robust nanoparticles, which could be recycled up to 98% and largely produced via wet chemistry. So, by implementing these recyclable nanoparticles on a 1 m^2^ area, 1.2 kg of seawater on average could be refined upon natural sun exposition within 1 h.^[^ ^59^ ^]^ Ozin et al. (2017) also presented his work in which germanium (Ge) nanocrystals were used as photothermal materials with different size indications, their effect on photothermal activity and enlightened as effective agents to solar desalination.^[^ ^60^ ^]^ Chen, Meijie et al. (2016), introduced an Au and Ag NPs were prepared using a sodium citrate thermal reduction process. Photothermal efficiency of the Au‐Ag blended NPs (1.75–0.15 ppm) was reported up to 30.97%, which was almost equal to the arithmetic sum (30.91%) of the photothermal conversion efficiencies of the Au NPs (1.75 ppm) and the Ag NPs (0.15 ppm), because of the separate absorptions of different wavebands by Au and Ag.^[^ ^61^ ^]^ Zhou, Lin et al. (2016) demonstrated light‐absorbing metallic nanostructures that are prepared by the PVD of gold deposition on different pore–sized alumina nano porous templates. A highly efficient (≈99%), broadband (200–10 mm) plasmonic light absorber was reported that it operates over the visible‐infrared regime. Besides, an improved light‐absorbing capacity has been invented by employing logically fabricated nanostructures from bimetallic nanocomposites.^[^ ^62^ ^]^ Zielinski et al. (2016), reported the fabrication of Ag/Au bimetallic hollow mesoporous plasmonic nano shells, which resulted in an increased solar evaporation efficiency as compared to its bulk solid counterparts.^[^ ^63^ ^]^

### 2.3 Nanoenabled Metal Oxide‐Based Absorbers

Metal oxides type semiconducting materials have been widely established as a suitable photothermal agent owing to highly tunable energy band and natural thermalization process.^[^ ^63^, ^64^ ^]^ Excitons formation is caused by the absorption of higher energy photons due to different bandgap engineering, called electron–hole pairs. These relaxations of electrons holes pairs occurs at the verge of the valence and conduction bands before recombining by converting the irradiative energy into heat.^[^ ^65^ ^]^ The photothermal conversion efficiency of these materials relies upon the bandgap because energy could be lost by emission as a result of direct electron–holes recombination across the bandgap. About 50% of the sunlight spectrum contains infrared radiation. Thus, light‐absorbing materials must occupy their absorption range lying in the infrared regions. In this regard, researches are being conducted to enhance the light‐absorbing capacity employing heteroatom doping through modulation of energy bandgap.^[^ ^66^, ^67^ ^]^

**Figure 5:**
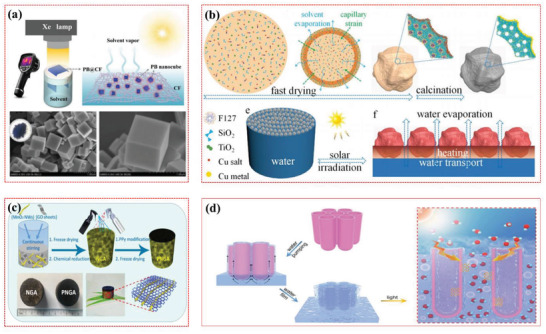
a) Represents the graphical representation of Prussian based nanocrystal structure for steam generation, and FESEM images show the nano cubical structure. Reproduced with permission.^[^ ^68^ ^]^ Copyright 2019, The Royal Society of Chemistry. b) complete step by step schematic demonstration of MPE‐xCu%/CST microparticles synthesis along with solar‐driven evaporator prototype. Reproduced with permission. ^[^ ^69^ ^]^ Copyright 2019, The Royal Society of Chemistry. c) MnO_2_ NWs induced reduced graphene/chitosan aerogel fabrication for a steam generation. Reproduced with permission.^[^ ^70^ ^]^ Copyright 2019, The Royal Society of Chemistry. d) the unique working principle of the solar steam generator using a 3D water membrane. Reproduced with permission.^[^ ^71^ ^]^ Copyright 2019, The Royal Society of Chemistry.

Qile et al. (2019) documented the preparation of cubic Prussian blue (PB@CF) nanocrystals were based on a stable photothermal device. Fabricated PB@CF composite was reported for outstanding optical absorption, photothermal transformation efficiency, and self‐pumping capacity of solvent for the removal of organic impurities with a rejection high up to 99.9%. Photothermal material increased the solar evaporation rate considerably up to 4.0–11.5 times comparative to without photothermal material, as demonstrated in **Figure** **5**a.^[^ ^68^ ^]^

Zhang et al. (2019) developed TiO_2_ and SiO_2_ based monodispersed, self‐floatable microparticles with spectacular structural and surface composition engineered by nanotech, as shown in Figure 5b. The mass‐specific evaporation rate of water was recorded up to 30.0 kg m^−2^ h ^−1^ g ^−1^ with stable water evaporation of ≈1.5 kg m^−2^ h ^−1^ and conversion efficiency up to ≈92.2% under one sun irradiation.^[^ ^69^ ^]^ Zhang, Zheng et al. (2019) reported that the fabrication of MnO_2_ based novel monolithic aerogel (PNGA) nanowire was reported as demonstrated in Figure 5c. Under 1 kW m^−2^ illumination intensity, photothermal efficiency recorded up to 93.8% which is almost 4.9 times higher compared to pure water (19.1%) along with good mechanical stability and outstanding filtration ability for ionic and other impurities with almost unaltered efficiency after conducting ten tests consecutively.^[^ ^70^ ^]^ Chen et al. (2017) prepared self‐ordered hydrophobic microspheres based on Fe_3_O_4_, Mn Fe_2_O_4_, Zn Fe_2_O_4_, and Co Fe_2_O_4_ capable of self‐floating on the water for solar thermal evaporation.^[^ ^72^ ^]^ Shi et al. (2018) introduced the fabrication of Cu Cr_2_O_4_ loaded quartz glass fiber membrane to be an excellent thermally stable photothermal device that has outstanding salt filtration potential with excellent photothermal efficiency and desalination ability.^[^ ^73^ ^]^ Titanium oxide is being used extensively for solar‐energy harvesting, owing to its energy‐level configuration compatibility with the solar spectrum and comparatively cheap to other metals. TiO_2_, a typical photo responsive semiconductor, has an intrinsic bandgap of ≈3eV, which corresponds to short‐wavelength light (< 400 nm, ultraviolet).^[^ ^74^, ^75^ ^]^ In fact, for effective absorption of solar energy, material bandgap should be narrow because most of the solar energy lies in this range. Li, Y. et al. demonstrated the light absorption of Ti_2_O_3_ over a wavelength range of 500–2500 nm, owing to its ultra‐small bandgap of ≈0.09 eV.^[^ ^76^ ^]^ An absorption level up to (92.5%) obtained with reflecting capacity of less than 10%. Doped semiconducting absorbers of desired bandgap referring to heat absorption, enabling steam generation upon the exposition of solar light with an intensity of 1 kWm^−2^ (one‐sun irradiance).^[^ ^76^ ^]^ Zhu et al. (2016) fabricated Titania nanocages based light‐capturing device possessing high optical absorption capacity for thermal conversion.^[^ ^77^ ^]^ Juan and Miaomiao et al. developed TiO*_x_* (*x* < 2), and Ti_2_O_3_ nanoparticles reported for efficient thermal absorption capacity, and their potential usage for solar absorption and desalination was demonstrated.^[^ ^78^, ^79^ ^]^ In 2019, Zr–30Ti alloy based anodized highly assembled strictly erect ZrO_2_ nanotubes arrays were fabricated. Prepared Zr(Ti)O_2_ membranes with interconnected super hydrophilic water channels (Figure 5d) are reported for high optical absorption and thermal conversion leading to an evaporation rate of 1.64 kg m^−2^ h^−1^ when illuminated under one sun at 20.5 °C.^[^ ^71^ ^]^

### 2.4 Nanoenabled Polymer‐Based Absorbers

Polymers have several advantages over their inorganic counterparts regarding their flexibility and easy moldability. Besides, stable and efficient water‐compatible photothermal polymers covering a wide range of solar spectrum are being paid less attention comparative to rare inorganic photothermal materials.^[^ ^14^, ^20^, ^25^, ^28^, ^80^, ^81^ ^]^ Conjugated polymers consist of π‐conjugated backbones of sp2 ‐hybridized carbon. That hybridization offer split energy levels, and a manipulated bandgap same as of inorganic semiconductors, ultimately leading towards high light absorption capacity.^[^ ^82^, ^83^ ^]^ Nevertheless, a given weighted conjugated polymer with a specific structure exhibits a special energy‐band configuration which causes to limit the absorption bandwidth, which in turn reduces the full‐spectrum sunlight power conversion efficiency.^[^ ^84^ ^]^

**Figure 6:**
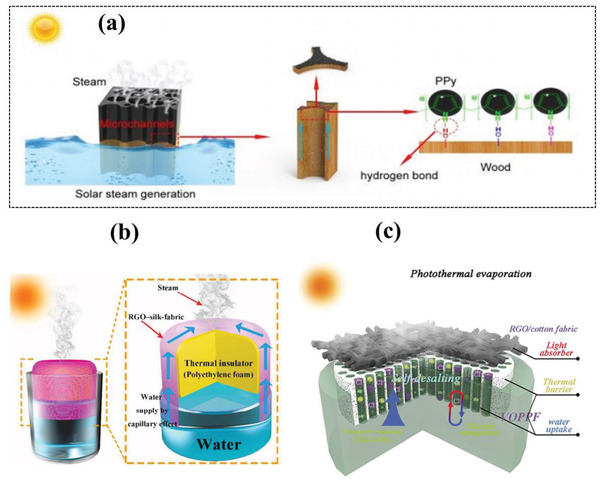
a) Polypyrrole coated natural wood emerged as an efficient solar steam generator for clean water production. Reproduced with permission.^[^ ^85^ ^]^ Copyright 2019, The Royal Society of Chemistry. b) rGO/Silk fabric‐based steam generator. Reproduced with permission.^[^ ^45^ ^]^ Copyright 2018, The Royal Society of Chemistry. c) Self‐salt resistant cotton fabric/rGO shows tremendous hydrophilicity and vertically oriented (VOPPF)water channel for a continuous steam generation along with self‐dissolving salt ability. Reproduced with permission.^[^ ^24^ ^]^ Copyright 2019, The Royal Society of Chemistry.

In 2019, polypyrrole decorated wood (PPywood) for solar evaporation enhancement was reported (**Figure** **6**a), which improved the wood light absorption, leads towards PPy‐wood high absorbance (> 90%) expanded over a wide range of wavelength from (300–2500 nm) that lies between ultraviolet region to the edge of the infrared region.^[^ ^85^ ^]^ In 2018, reduced graphene oxide and silk fabric (RGO–silk‐fabric) based photothermal device fabrication was reported as demonstrated in Figure 6b, with optical absorption range from 200 to 2500 nm making it a potential solar‐driven device. Besides, the rGO‐silk fabric demonstrated excellent washing ability, flexibility, and remarkable electrical strength for cheap, durable, and portable applications of solar steam generation.^[^ ^86^ ^]^ In 2019, reduced graphene oxide (RGO)/cotton fabric‐based unique solar evaporative system was fabricated with porous polyacrylonitrile foam (VOPPF). The reported system was vertically aligned enabling its self‐desalination as illustrated in Figure 6c. RGO/cotton fabric based device also showed strong salt rejecting properties with no apparent collection of salts that occurred during 12 h of simulated seawater (3.5 wt% NaCl) desalination on the top surface in simulated seawater. when 2g NaCl was placed on the top surface, it was completely passed through water channels down the water within 90 min.^[^ ^24^ ^]^ Han et al. (2020) introduced the fabrication of two‐fold evaporator structure with super hydrophilicity by joining through 3D porous hydrophilic carbon nanotubes planted on a glass microfiber membrane was reported. Which facilitates the efficient thermal localization on the surface area, ultimately leading towards a high evaporation rate of 1.62 kg m^−2^ h^−1^ along with thermal conversion efficiency of 87% under one sun intensity.^[^ ^87^ ^]^ In 2018, dark PPy coating deposited on different substrates by employing chemical vapor deposition polymerization (CVDP) technique, facilitating the efficient light absorber for effective collection and conversion of light to thermal energy for steam generation from solar‐driven interfacial water, was reported. Prepared PPy‐coated membranes were claimed for excellent harvesting of light with maximum stagnation temperature up to 82.3 °C under one sun illumination (1 kW m^−2^). Furthermore, the water evaporation could accelerate to 1.41 kg m^−2^ h^−1^, corresponding to 81.9% solar conversion efficiency, much relatable to the results of the state‐of‐the‐art photothermal membranes (**Table** **1**).^[^ ^88^ ^]^

**Table 1:** A comparative profile of nanoenabled photothermal materials, including carbon‐based, precious metals NPs, metal oxide NPs, and conjugated polymers

Material	Evaporation rate	Power density	Limitation	Reference
RGO+ MWCNTs	1.31	1	Expensive/	^[^ ^89^ ^]^
Carbon Sponge	1.39	1	Low efficiency	^[^ ^42^ ^]^
Carbonized foam	1.27	1	High temperature	^[^ ^90^ ^]^
Au/Ag	1.4	1	Expensive	^[^ ^91^ ^]^
Cu NPs	2.3256	2	Low efficiency	^[^ ^92^ ^]^
Black Ag	1.38	1	Expensive	^[^ ^93^ ^]^
WO_2.7_ /PLA	3.81	2.94	/	^[^ ^94^ ^]^
MoO*_x_* HNS	1.255	1	Complex	^[^ ^95^ ^]^
WO*_X_*	1.1017	1	Low efficiency	^[^ ^96^ ^]^
Gel+ AuF	1.356	1	Cheap	^[^ ^97^ ^]^
PPy nanosheets	1.38	1	Complex	^[^ ^98^ ^]^
APAC	1.2	1	Low efficiency	^[^ ^99^ ^]^

## 3 Photothermal Conversion Mechanisms

Solar energy is a huge energy source that could be transformed into several types of energy forms, through photovoltaic, photothermal and photochemical processes into chemical energy, electric energy, and thermal energy, respectively.^[^ ^57^, ^96^, ^100^, ^101^, ^102^, ^103^, ^104^ ^]^ Photothermal phenomena are one of the efficient processes that facilitates the maximum conversion performance possible. Thermal energy is created via thermal effect, which is caused by photo excitement phenomena in inorganic materials, such as carbon‐based materials, semiconductors, noble organic materials, dye esthetic, and conjugated polymers.^[^ ^16^, ^27^, ^35^, ^36^, ^60^, ^78^, ^83^, ^94^, ^105^, ^106^, ^107^ ^]^ The use of nanostructured materials as solar light collectibles is an emerging approach to use the solar energy in various applications, including the production of steam, the domestic heating of water, desalination, purification, distillation, and photothermal catalytic.^[^ ^42^, ^108^ ^]^ The major positive aspect of nanostructured materials is its heat modulation accuracy in the nanoscale in a specific area. Nanomaterials exhibit localized plasmonic resonance surfaces, quantum containment effects, and other exciting anomalies due to their unusual electronic and optical properties.^[^ ^1^, ^6^, ^109^, ^110^, ^111^, ^112^, ^113^, ^114^, ^115^, ^116^, ^117^ ^]^ The photothermal functions are assisted by these characteristics combined with wide surface area, adjustable surface properties, and tunable structures. The optical absorption and its efficient transformation into thermal energy cause the whole photothermal output in the development of active solar thermal collecting materials. Namely, bulk heating or localized plasma heating of metals, interfacial heating or non‐radiative semi‐conductor relaxation, and molecular thermal vibrations, based on various interaction mechanisms between electromagnetic radiation and matter.^[^ ^4^, ^6^, ^13^, ^14^, ^16^, ^17^, ^19^, ^29^, ^58^, ^61^, ^73^, ^91^, ^92^ ^]^

**Figure 7:**
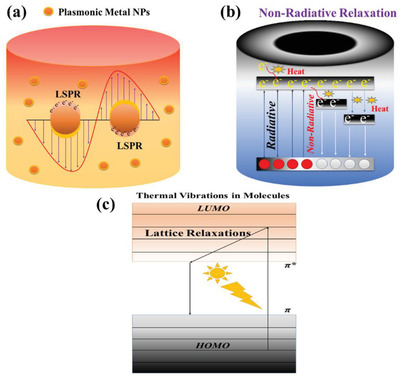
a–c) Photothermal conversion mechanisms. a) plasmonic metals followed by localized surface plasmon resonance (LSPR). b) metal oxide nanoparticles that are followed by non‐radiative relaxation behavior through defect states. c) π‐ π* bonds transition from HOMO to LUMO band, lead towards photothermal conversion in thermally vibrated molecules.

Electromagnetic radiation absorption in metallic materials is primarily caused by the intra‐band transitions when electrons are excited in the conduction band into higher energy states. Radiation absorption is called free carriers’ absorption by this process. The optical “skin range” for plasmonic metallic materials is about 10 nm where electromagnetic fields caused by thermal radiation which is mainly produced due to photothermal effect are at the surface plane.^[^ ^118^ ^]^ Due to the localized surface resonance (LSPR), some metallic nanoparticles transmit bright colors caused by wavelength extinction such as gold and silver.^[^ ^119^ ^]^ LSPR is a consistent charge oscillation‐based photon‐induced resonance when the photon frequency corresponds to the natural frequency of the metals surface electrons. Three successive phenomenal changes occur due to LSPR, nearfield magnification, hot electrons, and photothermal conversion.^[^ ^100^, ^117^ ^]^ This photothermally activated plasmonic resonance is a comparatively new area of interest, which began in 2002 primarily for medical use, e.g., photothermal cancer therapy.^[^ ^109^, ^117^ ^]^ Before the evaluation of this technology, plasmonic nanoparticles based heat generation was always followed by side effect.^[^ ^120^ ^]^ The plasmonic photothermal effect happens by the external illumination of metallic nanoparticles at a wavelength corresponding to their resonance frequency. This results in electronic oscillation by the excitation of electrons occurring at unoccupied states from occupied, which eventually contributes to a heat distribution.^[^ ^121^ ^]^ Either radiative emission or electron‐electron interactions lead to carrier replication, which causes the decay of these hot electrons.^[^ ^117^ ^]^ The decline by electron‐electron dispersing allocates the thermal electron energy, which causes the localized metal surface temperature to increase rapidly, as illustrated in **Figure** **7**a.^[^ ^122^, ^123^ ^]^ The change in localized temperatures causes equilibrium cooling leading towards electron‐photon energy transformation..^[^ ^69^ ^]^ Phonon‐phonon coupling causes the lattice cool down and discharges heat to the surrounding medium.^[^ ^120^ ^]^ The surface plasmon band shape and exact location will be affected by several features, such as particle size structure of material,^[^ ^122^ ^]^ dielectric medium constant,^[^ ^123^, ^124^ ^]^ and electric charge distribution among nanoparticles.^[^ ^124^ ^]^ Mainly, plasma resonance energies are responsible for heat flow from the hot carriers produced in plasmonic materials.

The optical absorption reveals a massive difference in wavelength near the bandgap energy in semiconducting materials. If a semiconductor is lightened, electron–hole pairs are formed with bandgap‐like energy.^[^ ^118^, ^125^ ^]^ The excited electron finally returns to low‐level states by transferring heat to impurities/defects or surface dangling bonds,^[^ ^106^ ^]^ either by radiative relief in the form of photons or by non‐radiative relaxation in the form of phonons (heat), as illustrated in Figure 7b. If energy is released as photons, this leads to a localized heating effect in the lattice, which overall distributes the temperature based on optical absorbing capacity and recombining properties for the bulk/surface region. The photothermal effect is caused by the optical absorption‐based excited diffusion followed by the recombination of electron–hole pairs, which causes temperature distribution in the material.^[^ ^126^, ^127^, ^128^, ^129^, ^130^ ^]^

Several organic materials are capable of absorbing light energy and transforming it into heat by vibrating atoms in a material. Mostly single‐bonded carbon atoms such as C—C, C—O, O—H, and C—H, possesses high differences in energy between σ and σ*, which are lower than 350 nm wavelength in the solar spectrum. Under solar irradiation, transition from σ to σ* is not achievable.^[^ ^13^, ^14^, ^16^, ^17^, ^28^ ^]^ In contrast, Pi (π) bonds typically possess fewer binding energies than σ‐bonds because of weakly bound electrons and are unable of getting excited with insufficient input energy from π to π* orbital. Besides, a redshift in the absorption spectrum could be caused by the conjugated bonds. The energy difference between the highest occupied molecular orbital (HOMO) and the lowest unoccupied molecular orbital (LUMO) decreases with an increasing number of π bonds. Moreover, electronic level spacing greatly affects the wavelength of these bands. Many conjugate π bonds in graphene‐like allotropes allow the excitation of electrons more comfortable with nearly every wavelength of solar radiation, coupled with different transitions of π‐ π* bonds, which enables them to a dark‐colored substance.^[^ ^131^, ^132^, ^133^, ^134^, ^135^, ^136^ ^]^ If the material is energized via illumination to a potential electronic molecule transition, enough energy absorption creates the possibility for an electron that could be excited from ground (Homo) to a higher energy orbital (LUMO), as shown in Figure 7c. By the application of electron machine, excited electrons relax and, subsequently, the light energy absorbed is transferred from excited electrons into the vibratory modes all over the lattice atoms, leading to a macroscopic rise in the material temperature.^[^ ^137^ ^]^

## 4 Efficient Heat‐to‐Steam Conversion and Vapor Condensation

Conversion from heat to steam is a highly complex process for clean water production. The practical approach for clean water production could be accomplished via seawater desalination by copping these certain challenges such as smooth water transport, salt deposition in water channels or photothermal surface layer, and steam condensation. The detailed discussion and theoretical analysis regarding the above issues have been investigated in this section.

### 4.1 Designs for Solar Thermal Capturing

The solar‐driven evaporation process has been extensively applied in the living and industrial side from the beginning as a primary thermodynamic mechanism.^[^ ^102^, ^138^, ^139^ ^]^ Depending on how the photothermal material is in the air, the solar system can be categorized into three stages: normal bottom thermal evaporation, evaporative suspension, solar‐driven interfacial evaporation (SDIE).^[^ ^17^ ^]^ Silver‐based solar stills followed by a traditional underfloor heating evaporation system, that improves the absorption of light using a blackish bottom layer for clean water storage system.^[^ ^140^ ^]^ Indeed, the efficiency of this evaporation system is comparatively small (30‐45%), the produced heat could not be utilized for steam generation directly due to bulk water.^[^ ^141^ ^]^ To minimize the heat dissipation of bulk water, the suspension device generates vapor through the dispersion of metallic plasmon, carbon, and other nanoparticles induced into the fluid, which enhances the light absorption and lowering thermal resistance, effectively improves the evaporation efficiency.^[^ ^57^, ^142^ ^]^ Until now, the suspension system's evaporation mechanism remained a debatable issue. Han et al. assumed that when the nanoparticle absorbs light following the decreased thermal conductivity of the metal‐liquid interface,^[^ ^143^ ^]^ it creates a difference in temperature between the surroundings and induced nanoparticles. This rise in local temperature produced vapor insulation on the nanoparticle surface. The vapor cladding expands extensively under continuous illumination extended and eventually left the water body by resilience.^[^ ^144^, ^145^, ^146^ ^]^ In order to produce nano‐fluid heating and steam generation by traditional overall heating of the suspension fluid, Chen et al. established therapeutic transfer models on behalf of numerical investigations.^[^ ^145^ ^]^ In 2020, Moh and Wen et al. further noted that the first stage of steam production was primarily due to the extremely variable temperature and radiant energy distribution, which resulted in the partial boiling of a super‐heated area and its vaporization.^[^ ^49^, ^147^ ^]^

Irrespective of the suspension procedure of the vaporization mechanism, it's quite unavoidable that heat radiation, conduction, and convection would spread the transferred thermal energy of photothermal materials into the non‐evaporative component, resulting in a reduction in distributors’ evaporation effect.^[^ ^63^, ^148^ ^]^ Besides, high costs and inhomogeneity are critical challenges in practical applications in the recycling of nanoparticles.^[^ ^149^ ^]^ In 2019, Luo et al. proposed an innovative work to minimize excessive heat from bulk water to improve evaporation effectiveness with a carbon‐based double layer used in solar steam production via heat localization.^[^ ^150^, ^151^ ^]^ Researchers have carried out extensive work in the last five years regarding interfacial investigation between solar steam generation, photothermal conversion phenomena, water pathways, water enthalpy, and improvised thermal management. The improvization of thermal management has been accomplished using an interfacial evaporation system in which the photothermal layer is linked with bulk water via capillary microchannel water channels instead of direct contact. Transmitted thermal energy is continually absorbed at a water‐air interface to heat water molecules, minimizing thermal energy transfers to bulk water. This selective method of heating achieves over 90% vaporization efficiency without optical concentrations with the introduction of various new structures.^[^ ^152^, ^153^ ^]^

### 4.2 Heat Conduction

If the solar absorber is not insulated and direct interaction to bulk water, solar evaporator device disperses of its energy through heat conduction into bulk water that is the major component of the total heat loss.^[^ ^154^ ^]^ Thus, heat loss is a very crucial challenge for a solar‐driven evaporation system to suppress it. Fourier heat control rule could be generalized to quantify conduction heat loss, followed equation narrates the heat conduction loss

(1) $$\begin{array}{c} \phi =-A\times h_{\text{cond}}\times \frac{\text{d}T}{\text{d}x} \end{array}$$

(2) $$q_{\text{cond}}=h_{\text{cond}}\left(T_{\text{Insulator},\;\text{upper}}-T_{\text{Insulator},\;\text{bottom}}\right)\times 1\;\text{d}$$

ϕ indicates the conduction heat flux (w), *dT*/*dx* represents the temperature gradient of insulator between top to the bottom surface (Km^−1^), denotes the top surface area of an insulator (m^2^). The above equation shows that heat conduction losses purely rely on temperature gradient (*dT*/*dx*) and thermal conductivity (*h*_cound_), respectively. Indeed, minimum thermal conductivity may lead to efficient heat accumulation that could be achieved by introducing new thermal insulating designs.

The development of microporous structural designs is a natural approach to deal with heat losses and reduces thermal conductivity. In such types of structures, thermal conductivity is decreased by utilizing air as a thermal barrier such that by enhancing the porosity, the thermal conductivity is reduced. In 2018, Qu et al. reported the fabrication of a highly vertically ordered pillar array of the graphene‐assembled framework (HOPGF) to acquire the lowest thermal conductivity of 18–35 mW m^−1^ K ^−1^ and a high‐water evaporation rate of 2.10 kg m^−2^ h^−1^.^[^ ^155^ ^]^ Zhu et al. (2017) fabricated a GO‐based aerogel along with the free‐floating ability and also low thermal conductivity of 0.0047–0.035 W m^−1^ K ^−1^.^[^ ^44^ ^]^ Thermal insulating efficiency of the material could be degraded during evaporation at water‐insulating pores interface that resulted in unexpected values. In this regard, in 2019, Liu et al. proposed the fabrication of polypyrrole (PPy) coated pre‐pressed melamine foam. The polypyrrole layer of this material showed the lowest thermal conductivities of 0.06 and 0.48 W m^−1^ K^−1^ in a dry and wet state, respectively.^[^ ^156^ ^]^ Thus, thermal conductivity in the wet state should be considered as an efficient thermal conductivity during evaporation, thus maximizing the material's water permeability to achieve as little thermo‐conductivity as possible.

**Figure 8:**
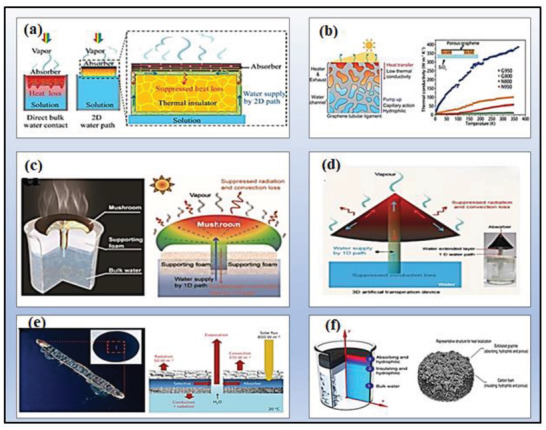
a) Schematics of devices of SDIE with suppressed heat loss and 2D water supply. Reproduced with permission.^[^ ^149^ ^]^ Copyright 2016, the National Academy of Sciences of USA. b) Schematic of the solar evaporation process of 3D porous graphene sheets with nitrogen doping and thermal conductivity of porous graphene samples. Without nitrogen doping at different growth temperatures. Reproduced with permission.^[^ ^157^ ^]^ Copyright 2015, Wiley‐VCH. c) Schematic of a mushroom‐based solar evaporation device and the heat behavior under one sun. Reproduced with permission.^[^ ^165^ ^]^ Copyright 2017, Wiley‐VCH. d) Schematics of 3D artificial transpiration device and optical image (inset). Reproduced with permission.^[^ ^154^ ^]^ Copyright 2018, Oxford University Press. e) Ambient steam generator composed of spectrally selective copper along with energy and heat transfer. Reproduced with permission.^[^ ^168^ ^]^ Copyright 2016, Springer, Nature. f) double‐layer structure with cross‐view of temperature distribution along with the optical image. Reproduced with permission.^[^ ^150^ ^]^ Copyright 2014, Springer, Nature.

A thermally isolated system, which can eliminate the evaporation hotspot from the bulk water, is an effective way to avoid the conduction loss. To date, effective insulation via heat barrier and water transport was mainly achieved with the creation of bifunctional channels such as 1D and 2D water pathways.^[^ ^157^ ^]^ The water channel optimization reduces heat conduction loss and accumulates heat within the matrix, and it will lead to continuous steam generation. The water channel optimization reduces heat conduction loss and accumulates heat within the matrix, and it will lead to continuous steam generation. In 2016, Wang et al. developed a novel double‐layer structure for efficient solar steam generation system (**Figure** **8**a), in which reduced graphene oxide (rGO) based photothermal sheets was utilized as a top surface and the porous mixed cellulose esters (MCE) membrane used as a bottom supporting layer. The followed photothermal system along with polyethyleneimine could absorb efficient solar energy and converted into heat at a water‐air interface. The efficient evaporation efficiency (≈60%) was reported under only 1 kW m^−2^ irradiation.^[^ ^149^ ^]^ Whereas, Chen et al. (Figure 8b) also presented the fabrication of a nitrogen‐doped porous graphene sheet carrying a reduced thermal conductivity of 9.0 ± 1.2 W m^−1^ K ^−1.[^ ^157^ ^]^ Afterward, there were studies on a variety of comparable two‐layer structure. such as air‐laid paper,^[^ ^108^, ^158^ ^]^ cellulose,^[^ ^107^, ^159^ ^]^ AAO,^[^ ^56^, ^62^ ^]^ leaf,^[^ ^160^ ^]^ wood^[^ ^58^, ^161^ ^]^ carbon foam,^[^ ^162^ ^]^ geopolymer^[^ ^105^ ^]^ and electro spun polyacrylonitrile^[^ ^104^ ^]^ were utilized as the bottom materials for water transport and insulation. The observed low efficiency of this structure is not enough due to weakening the thermal barrier as water entering the pores of the evaporation system.

The development of vertically oriented 2D water channels enabled continuous steam generation and reduces heat losses due to the separation of the photothermal layer from bulk water instead of double‐layer evaporation structure as prescribed above. In 2016, Xiu et al. also contributed to design a thermally insulated system in which graphene oxide (GO) film was confined in the 2D water path. To achieve the lowest thermal conductivity, GO film was kept separated from bulk water and polystyrene foam was wrapped by cellulose. The decoration of cellulose may reduce thermal losses and also improvise photothermal conversion efficiency up to 80% under 1 kW m^−2^.^[^ ^163^ ^]^ Liu et al. (2019) documented the manufacturing of polystyrene‐based reformed carbon fibers, which showed thermal conversion efficiency of 92.5% (1 kW m^−2^) with very minute heat conduction loss of 3%.^[^ ^164^ ^]^ The 1D water channel influenced by transpiration may cause a reduction in heat conduction loss and improvise the water channel matrix also. In 2017, Zhu et al. proposed the fabrication of carbonized mushroom (Figure 8c), which exhibits the efficient absorption of 78% solar for a steam generation with 0.2% heat conduction losses.^[^ ^165^ ^]^ In 2018, Miao et al. stated his contribution by fabricating carbonized natural wood‐based photothermal device which was implanted on an extended polyethylene foam and air‐laid paper wick aiding in solar absorption, that reported 91.3% (1 kW m^−2^) efficiency for a steam generation with 4.5% heat conduction loses.^[^ ^166^ ^]^ Huang et al. (2019) reported a photothermal evaporating system that is composed of a carbon‐black/cellulose‐sponge system, which accomplished 91.5% (under 1 kW m^−2^) rate of vaporization with very minute heat conduction loss of 0.714%.^[^ ^167^ ^]^ While water channel optimization greatly enhances the vaporization rate with a reduction in conduction loss, salt deposition problems should be minimized. The equilibrium between salt accumulation and water production will be discussed later.

### 4.3 Heat Radiation

The top interfacial solar‐driven evaporator surface is mainly based on heat losses caused by heat convection and radiation. Heat loss can be significant, particularly at high temperatures. Stefan‐Boltzmann law could be generalized to calculate heat radiation loss

(3) $$\begin{array}{c} q_{\text{rad}}=\varepsilon_{\text{eff}}\sigma T_{\text{absor}}^{\;4}-\varepsilon_{\text{ambient}}\sigma T_{\text{ambient}}^{\;4} \end{array}$$

where ε_ambient_ represents surrounding environment thermal emissivity. The use of *T*_ambient_ in state‐of‐the‐art literature is currently controversial because steam and water are semi‐transparent to heat radiation.^[^ ^101^ ^]^ In this context, few people give preference to room temperature selection,^[^ ^49^ ^]^ whereas others consider it due to the hot steam and water layer above the photothermal materials, the top steam temperature should be selected.^[^ ^169^ ^]^ The choice of the temperature could be random, ε_eff_ < ε_ambient_ · <1. Therefore, equation (3) can be simplified to the following

(4) $$q_{\text{rad}}=\varepsilon_{\text{eff}}\sigma \left(T_{\text{absor}}^{4}-T_{\text{ambient}}^{4}\right)$$

The effective thermal_emissivity_ (ε_eff_) can be calculated as:^[^ ^170^ ^]^

(5) $$\begin{array}{c} \varepsilon_{\text{eff}}=\frac{M_{\text{absor}}\left(T_{\text{absor}}\right)}{M_{\text{bb}}\left(T_{\text{absor}}\right)}=\frac{\int \varepsilon \left(\lambda \right)\;E_{\text{bb}}\;\left(T_{\text{absor}},\;\lambda \right)\;d\lambda }{\int E_{\text{bb}}\;\left(T_{\text{absor}},\;\lambda \right)\;d\lambda \;} \end{array}$$

where (*T*_absor_) and (*T*_absor_) are radiation emission in form of spectral line for absorbing surface and standard blackbody under absorbing temperature. (λ) is for the thermal emissivity of the absorber, *E*_bb_ (*T*_absor_, λ) is the thermal irradiance of the standard blackbody. By Equation (5), effective thermal emissivity (ε_eff_) along with absorber temperature (*T*_absor_) directly influence the heat radiation loss.

The region of the thermal radiation window is specified by the surface temperature of the solar absorbers. Perfect solar absorbing materials are those, possesses high absorption of radiation expended over solar spectrum range from (280–2500 nm) along with lower thermal emission (2000 nm–25 µm) within the thermal window region, that leads towards the reduction of heat loss due to thermal radiation.^[^ ^171^ ^]^ These two properties are used for the field of solar‐driven interfacial evaporation (SDIE) systems along with spectrally selective absorbers. For example, Chen et al. suggested the fabrication of solar absorber with discrete spectral emission (cermet‐type absorber, α = 0.9 and ε_*eff*_ = 0.07) for generating 100 °C steam without employing any optical focusing device under ambient environment, which came out with very low thermal radiation based heat loss of up to 5%.^[^ ^22^ ^]^ There are specific difficulties with SDIE applications for example reduction in sustainability for resisting seawater,^[^ ^172^ ^]^ and the dense substrate avoids steam escapes and requires manual slot cutting in spectrally selective absorbers. By incorporating the membrane‐based solar distillation system, vapor flow is made downward forcefully by breaking down spectral absorber limits.^[^ ^15^, ^173^ ^]^

**Figure 9:**
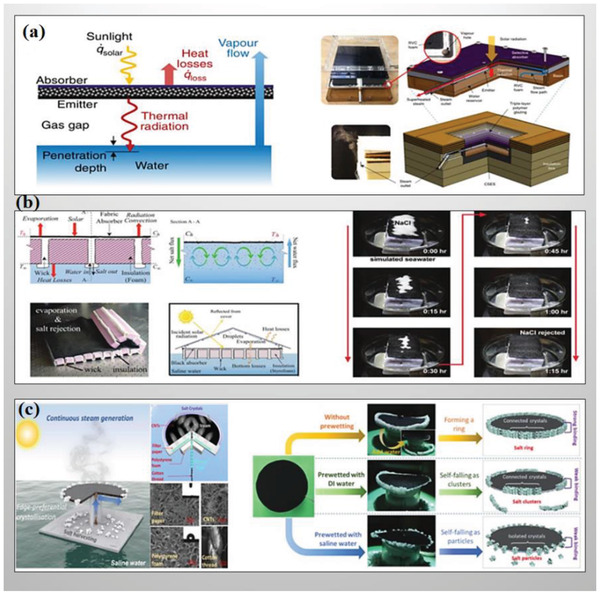
a) Schematic demonstration of energy transportation of contactless solar‐powered structure (left) and assembly structure (right). Reproduced with permission.^[^ ^175^ ^]^ Copyright 2018, Springer Nature. b) Schematic of integrated floating solar still with Simultaneous salt rejection by diffusion and advection, and heat localization ability. Reproduced with permission.^[^ ^183^ ^]^ Copyright 2018, The Royal Society of Chemistry. c) Schematic illustration of the novel design for continuous solar steam generation and salt harvesting (left). And the digital photographs of different crystallization state without prewetting, with DI water prewetting and saline water prewetting (right). Reproduced with permission.^[^ ^188^ ^]^ Copyright 2019, The Royal Society of Chemistry.

It also provides an efficient means by reducing the absorber surface temperatures through structural design, thereby minimizing thermal radiation. The increase of the absorber's active evaporation area can potentially lessen the absorber's temperature of vaporization. Zhu et al. achieved a high rate of vaporization up to 85% by fabricating an artificial 3D transpiration system which loses a very lower amount of heat 7% by thermal radiation, with a vaporization temperature of 32.7 °C.^[^ ^174^ ^]^ The structures are similar in design to carbonized mushroom,^[^ ^165^ ^]^ cup‐shaped structures in the 3D cylindric form,^[^ ^103^ ^]^ and the environmental energy‐enhancing interfacial solar steam generator^[^ ^175^ ^]^ (**Figure** **9**a). The above two steam generating systems are enabled to minimize various heat losses by lowering the vaporization temperature along with the capability of extracting atmospheric energy to break the theoretical limits in the efficiency and vaporization rate. The temperature of the outer wall should be lowered to reduce radiative heat loss, cup‐shaped along with the 3D cylindrical design that can utilize the internal wall for recycling heat loss that is caused by convection and radiation from the cup‐shaped bottom. Recently, Chen et al. reported the fabrication of a superfast vapor generating system through thermal radiations by employing a contactless evaporation device that is enabling of sorting out the fouling problem of absorbers.^[^ ^22^ ^]^

### 4.4 Heat Convection

Heat convection is a dynamic heat transmission process of thermal conduction plus fluid motion. Newton's law of cooling can calculate the heat convection loss

(6) $$\begin{array}{c} q_{\text{conv}}=h{\;}_{\text{conv}}\left(T_{\text{absor}}-T_{\text{ambient}}\right) \end{array}$$

According to the above equation, the convection heat transfer coefficient (*h*_conv_) is reduced to avoid loss of convection heat by lowering the temperature of surface temperature through structural design. *h*_conv_ is, therefore, a state value rather than eigenvalue that is influenced by several factors: 1) phase variations and fluid kind; 2) various features possessed by a fluid such as specific heat, viscosity, volume expansion coefficient and density; 3) fluid temperature; 4) flowing state of fluid; 5) reason behind the flow of fluid (natural convection, forced convection); 6) structural design, dimensions and position of surface transferring heat. Since heat convection seems hard for separate regulation, few types of research about controlling heat convection have yet been conducted. In this regard, Chen's group did exceptional work, which used a wrapping covering to transmit sunlight and mitigate heat loss due to convection.^[^ ^22^ ^]^ Decreasing convective space is also an option.

### 4.5 Water Transport and Salt Dissipation

The water path plays a substantial role in the steam generation process. The optimization of the water supply rate should be accurately matched with the evaporation rate for continuous steam generation. Desalination is one of the most effective strategies for raising the availability of freshwater beyond natural reservoirs of freshwater. The efficient utilization of renewable energy resources for seawater desalination is a popular tool for clean water production.^[^ ^6^, ^13^, ^14^, ^17^ ^]^ As compared to membrane technology, solar‐driven water evaporation is less susceptible due to large variabilities in experimental conditions, although highly contaminated water may contribute to the complicated process of desalination. However, its practical applications are constrained by its low efficiencies caused by reduced absorption of solar energy and excessive heat losses. Besides, efforts to improve its performance by using large optical concentrators along with thermal insulations are preventing its viability and scalability. A considerable increase in the production of solar absorbers has led to a significant rise in conversion efficiencies. Multiple methods have been used for solar desalination, ranging from scattered particle, solar absorber film to porous‐support structure.^[^ ^4^, ^6^, ^12^, ^13^, ^14^, ^68^, ^160^, ^176^, ^177^ ^]^

### 4.6 Designs of Water Pathway

Water transport in thermally activated evaporation field by solar energy is typically influenced by porous structure based capillary forces, which can be enhanced by top negative pressure from evaporation. At the start, hydrophobic membranes or simple absorber devices were employed as steam generators in the early evaporation devices (set directly on the surface of the water, without complex thermal insulation).^[^ ^178^, ^179^ ^]^ The former is not equipped to convey water; the latter offers water by the capillary action of its porous structure. The bifunctional, 2D, and 1D waterways were subsequently introduced for an optimal balance between loss of heat conduction and water transportation. Hu et al. proposed the utilization of interconnected, naturally made 3D micro‐/nanochannel for water evaporation devices from a carbonated wood evaporation system.^[^ ^180^ ^]^ Zhu et al. suggested the fabrication of 2D and 1D water channels composed of cellulose paper‐based folded polystyrene foam commercial cotton pole respectively and employed them for photothermal materials separated physically from the water body.^[^ ^163^, ^174^ ^]^ There have been documented several analogous structures regarding water pathways; for example, Liu et al. proposed the construction of a 2D water pathway for desalination from a nonwoven cotton fabric simulated by carbon fibrous device. They investigated the influence of the number of water channels on the vaporization efficiency rate.^[^ ^181^ ^]^ Bai et al., 2019, proposed the fabrication of aerogel based on polyacrylamide possessing a 3D radial and centrosymmetric structure to accomplish anti‐gravity rapid and large‐spaced transport.^[^ ^182^ ^]^ As the transfer of water is in direct incorporation with energy, more work is required to approach efficiently the water production and the rate of vapor generation and reduction of heat conduction losses along with the prevention of salt accumulation.

### 4.7 Theoretical Analysis for Salt Deposition

During the vapor production cycle, the salt concentration of the evaporating surface is gradually increasing. Meanwhile, migration of salt ions through water pathways again into the bulk water due to gradient of concentration, until diffusion rate and salt accumulation rate achieve an equilibrium state, at this point deposition of salt occurs. Both diffusion and advection are prominent phenomena of assisting salt.^[^ ^183^ ^]^ Here, we rely primarily on diffusion for the conservative measure. The rate of salt accumulation of the evaporation surface shall, to avoid deposition of the salt, be less or equal to salt back diffusion rate in the steady‐state bulk water. By considering Fick's diffusion law, the following equations could be written as

(7) $$a_{\text{salt,}\;\text{acc}}=\frac{V_{\text{evap}}C_{\text{salt,}\;\text{bulk}}}{1-C_{\text{salt,}\;\text{bulk}}}$$

(8) $$a_{\text{salt,}\;\text{diff}}=\frac{D_{\text{Nacl}\rho \varphi }\left(C_{\text{salt,}\;\text{evap}}-C_{\text{salt,}\;\text{bulk}}\right)}{d_{\text{water}}}$$

Where α_salt, acc_, and α_salt, diff_ represents the accumulation or diffusion rate of salt, *C*_salt_, and *C*_salt_, are salt mass fraction at evaporating layer or underlying water body. *D*_Nacl_ is the diffusion coefficient of NaCl (7.16 × 10^−6^ m^2^ h ^−1^), ρ is for seawater density (1.04 × 10^3^ kg m^−3^, using natural convection, salt diffusing back to the water body is dispersed), φ depicts water transferring material porosity, *d*_water_ represents water transferring material thickness.^[^ ^101^ ^]^ *C*_salt,_ is supposed getting saturated at (26 wt%), *C*_salt,bulk_, is local sea water concentration (3 wt%). After integration, we obtain the following equation

(9) $$a_{\text{salt,}\;\text{diff}\;\text{salt,}\;\text{acc}}=\frac{D_{\text{NaCl}\;\rho \varphi \;\text{hlv}\;\times \;23\;\text{wt}\%\;\times \;97\;\text{wt}\%}}{d_{\text{water}\;\eta \text{q}\;\text{solar}\;\times \;3\text{wt}\%}}\;\geq \;1$$

According to the equation, water passing material's thickness, porosity and the illumination of light directly influence the salt deposition. The ratio between the evaporating region and waterway area with the evaporation region in the 2D waterway structure is equal to the ratio between the diffusion rate and the evaporation rate.^[^ ^184^ ^]^ Though the reduction of thickness in water transport and the enhancement of porosity lead to salt rejection, thermal conduction losses are also improved and should be managed in the optimum range, and this process is called a complicated coupling process.

### 4.8 Salt‐Rejection Methods

In a steam generation, transporting the water is most concerning critical factors. The supply rate of water must be precisely matched with that of steam. That loss of heat is carried with when water amount is more significant, while insufficient water cannot satisfy the requirements for producing steam and causes the salt accumulation of salt on the absorber surface to influence the system's light‐absorbing capacity and water feed properties.^[^ ^23^, ^47^, ^104^, ^131^, ^153^, ^180^, ^183^, ^185^, ^186^, ^187^, ^188^ ^]^ In this section, the water transport route is explored, and theoretically, the effects of the deposition of salt are studied, and methods for salt repression in recent years are summarized. Despite significant development and improvements in material structural properties and the evaporation rate in SDIE in recent years, the broad application of aquatic desalination was inefficient. One of the big problems is the low stability of salt blocking.^[^ ^104^, ^131^, ^180^, ^186^ ^]^

Till now, there has been documented several common salt blocking approaches such as repeated washing, mechanical exfoliation, and dip‐squeezing,^[^ ^6^, ^189^ ^]^ but theses process cannot stand the manufacturing efficiency and also not much cost‐effective. To cope with this problem, there have recently been proposed four types of revolutionary methods. The first approach is to prevent salt through the hydrophobic interface in the lower water environment.^[^ ^190^ ^]^ In this context, Zhu et al. proposed the fabrication double‐layered Janus absorbers with top hydrophobic carbon black nanoparticles coating polymethylmethacrylate layer (CB/PMMA) and the bottom hydrophilic polyacrylonitrile (PAN) layer. During the desalination process, salt ions got captured in the PAN layer instead of being crystallized on the CB/PMMA layer,^[^ ^104^ ^]^ the stability of 16 days (1 kW m^−2^) was stated for prepared Janus absorber. Although this method can suppress salt accumulation efficiently, it does compromise the system's thermal insulation effect. The second method involves the dissolution of salt ions by diffusion and convection from the evaporating surface into the underlying water body.^[^ ^24^, ^181^, ^191^ ^]^ Hu et al. achieved the distillation rate of (15 wt%) for water salinity by surface‐carbonized bimodal porous balsa wood membrane. The characteristic feature of the unique 3D interlinked porous structure is rapid self‐pumping ability evaporated brine at substrate surface via diffusion convection and capillary pumping facilitating an increase in vapor generation rate. Excellent stability was achieved with almost unchanged efficiency of (1.7 kg m^2^ h^−1^) when putting under 2 sun illumination for 20 days (7 h each day).^[^ ^157^ ^]^

Chen et al. stated the manufacturing of solar activated device enabled of self‐floating with capable of simultaneously producing localized surface heat and salt rejection, black hydrophilic cellulose fiber was employed for constituting top layer acting as was solar radiation absorber, whereas bottom layer was composed of adjacent layers of the white fiber of extended polystyrene foam.^[^ ^183^ ^]^ The wick‐to‐evaporation ratio was calculated carefully to ensure the back diffusion of deposited salt ions into the underlying water body from the evaporating surface by diffusion convection. This fabricated device could not only resist salt accumulation continuously for seven days but can dissolve it back to the underlying bulk water body under one sun illumination. The heat position and the salt resistance could successfully obtain by controlling diffusion and convection, which is the most widespread technique currently used. The third approach regarding salt‐rejection and salt collection could be achieved simultaneously via structural design optimization. In this regard, Zhang et al. reported an innovative fabrication approach to separate salt crystals spatially from thermally activated devices to achieve edge‐preferential crystallization along with gravity‐assisted salt harvesting.^[^ ^188^ ^]^ Chen et al. also reported a solar‐powered steam generator that is not directly contacted with water, and the surface water was warmed via thermal radiation. This non‐contact evaporator enabled to prevent salt deposition in the absorber, and superheated steam was produced in a single sun without any pressurization.^[^ ^175^ ^]^

### 4.9 Vapor Condensation

The transparent cover is typically used for the condensation and collection of the sun's steam during the solar desalination cycle and thus for receiving freshwater supplies. The water production levels, which rely on the evaporation and collecting rate of condensate, are more intuitive than the rate of vaporization. Though the steam generation efficiency rate has reached a sufficiently high level at present, the condensation rate of water vapors is much lower and even ignored, that significantly block and limits its practical application.^[^ ^13^, ^15^, ^28^, ^192^ ^]^ Water film is formed by vapors or condensate collection from the transparent covering of the steam condensing device, causing optical loss, which directly influences the freshwater producing efficiency.^[^ ^193^ ^]^ Also, the temperature of the evaporative surface and the relative moisture of the device is improved for obtaining a high condensation rate comparative to the steam generation rate to prevent the vaporization of condensate.^[^ ^15^ ^]^

Chen et al. presented the fabrication of polyester covers substituted by glass covers enable of floating over water body which resulted in 35% optical loss.^[^ ^183^ ^]^ Elevation of water and hydrophobic polymer contact angle allows the development of a range of water droplets. Anyways, it's more apparent than the water film yet.^[^ ^177^ ^]^ So, the hydrophilic transparent polymer cover will decrease the optical loss and should have contact angles less than 50°.^[^ ^194^ ^]^ The glass cover is considered fragile and has a high alternative cost, although its light transmission and its water yield are more elevated. The polymer cover has traditionally been more realistic. Instead, changing the location of the condensate vapors is more efficient for eliminating the effect of condensate on transparent cover. For instance, for the transport of the vapor to the lower condensation chamber, Yu et al. documented the airflow use as a carrier gas, staggering the condensing position from the power input positions to remove condensing effect on light.^[^ ^195^ ^]^

**Figure 10:**
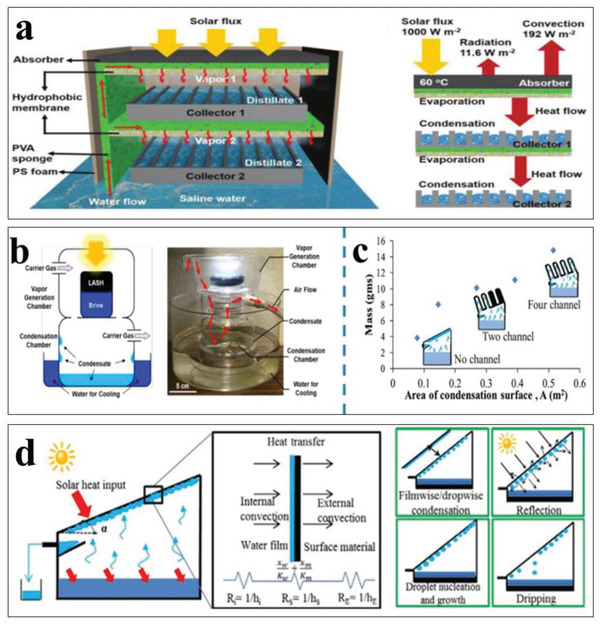
a) The cross‐sectional view and energy transportation schematic of solar‐thermal membrane distillation system. Reproduced with permission.^[^ ^173^ ^]^ Copyright 2018, Wiley‐VCH. b) Photograph of steam generation chamber separate from condensation. Reproduced with permission.^[^ ^195^ ^]^ Copyright 2019, American Chemical Society. (c) The increasing effect in the condensation area. Reproduced with permission.^[^ ^196^ ^]^ Copyright 2015. Elsevier. (d) Schematic illustration for typical condensation and heat transfer at the cover surface. Reproduced with permission.^[^ ^177^ ^]^ Copyright 2013, Elsevier.

Zhou et al. have developed and produced an integrated steam‐generating solar activated membrane that uses a superhydrophobic membrane with vertical channels to assist the downward flow of vapors and condensation, separately managing both processes of input energy and water collection, thus preventing condensation from affecting incident light.^[^ ^173^ ^]^ A decrease in condensation rate was observed as the transparent cover of the condensation increased due to an increase in temperature. When the condensation rate is inadequate, particularly for enclosed collecting systems of water, the efficiency of vaporization and the rate of water production are hindered in operations. Zhu et al. exhibited a reduction in the evaporation rate of 75% to 18% for half‐closed systems from open systems.^[^ ^159^ ^]^ Hence, by enhancing the condensation area, the output rate of water will also increase (**Figure** **10**).^[^ ^196^ ^]^

### 4.10 Water Purification

While high temperatures accelerate evaporation, they eventually evaporate and accumulate the undesirable residue as an unpurified extract. Whereas, solar energy can sufficiently evaporate water from source water and achieve evaporation point below the boiling temperature as well. Upon cooling, water vapor then condenses in the form of purified distillate. The obtained distillate is thus purified from most contaminants and impurities, releasing unnecessary traces in the remaining water such as minerals, heavy metals, microbial species.^[^ ^3^, ^138^, ^192^ ^]^ As a result, some scientists have struggled to pave the path for solar evaporation‐based water purification to eliminate several water pollutants simultaneously. To date, two different purification mechanisms, physical adsorption, and photocatalytic degradation of multiple pollutants have primarily been stated as efficient bifunctional solar evaporative systems for freshwater production.^[^ ^3^, ^138^, ^192^ ^]^

The carbon solar absorber material is a valid candidate for the combination of water evaporation and purification functions owing to its unique adsorption ability. For example, hollow carbon spheres^[^ ^197^ ^]^ have been investigated for fixed‐bed oil adsorption and solar evaporation. In contrast, carbon fabric^[^ ^198^ ^]^ and rGO film on air‐laid paper^[^ ^108^ ^]^ have been demonstrated for the effective removal of organic contaminants in industrial wastewater during solar steam generation. Because of their wide surface areas, ideal functional groups, and porosity of their structure, physical adsorption of these materials facilitates this purification mechanism. Moreover, Deng et al. show that pushing force created by the upward vapors flow near the water surface, the photothermal absorption is accelerated mainly by solar‐driven evaporation.^[^ ^199^ ^]^ This physical adsorption can also influence solar evaporation because the accumulated pollutants inside photothermal materials cause optical blockage along with decreased interface surface among photothermal material and water.^[^ ^3^, ^138^ ^]^

An alternative option is the introduction of photocatalysts in the solar dye content for eliminating photocatalytic contaminants through photocatalytic degradation. For efficiently proving the elimination of Rhodamine B (RhB) utilizing photocatalysis, TiO_2_ nanostructures were incorporated into the plasmonic^[^ ^200^ ^]^ and carbon^[^ ^198^, ^199^ ^]^ solar‐absorber materials. Solar absorbing material was deposited by TiO_2_ while solar evaporation generates. It captures the solar spectrum in the range of UV radiation along with electron–hole generation for RhB^[^ ^36^, ^201^ ^]^ degradation, whereas photothermal material for photothermic transformation for performing the steam generation is absorbed by the visible and NIR light.^[^ ^199^, ^200^ ^]^ This method for photocatalysis is also coupled with physical adsorption to eliminate contaminants effectively. In this scenario, contaminants would be driven to photothermal material having porous photocatalyst through solar steam on the water surface to get adsorbed, followed by the photocatalyst surface degradation. Besides, solar evaporation further purifies the distillate, and solar energy is the primary motive of this continuous, multi‐step purification cycle. However, the elimination of volatile organic compounds (VOCs) needs to be discussed in the field of purification.^[^ ^3^, ^138^ ^]^ Due to their low boiling points, VOCs in the water supply often can vaporize during solar evaporation and therefore remain in the distillate collected. Therefore, structures of photothermic materials should be further explored in the hypotheses of the removal of not only non‐solid organic pollutants but also VOCs through adsorption, photocatalysis^[^ ^202^, ^203^ ^]^ or photothermal‐assisted catalysis process for water purification.^[^ ^36^, ^201^ ^]^

## 5 Water‐Energy Nexus

Solar energy based photovoltaic, photochemical, and photothermal processes have extensive applications in real life as cheap energy sources and clean energy production. At the same time, solar energy‐based vaporization is a cheap energy source and an easy approach to meet all water and energy crises logically. Solar steam generation‐based energy production along with the accumulation of freshwater is a recently emerged energy harvesting area. Such research addresses the two global problems, namely the limited freshwater and fossil fuel reservoirs. With the simultaneous development in primary human needs, such as fuel source and free sunlight supply, enhances more photothermal conversion overall performance.^[^ ^13^, ^17^, ^28^, ^33^, ^36^ ^]^

Solar vaporization process‐based thermal energy/heat may be used directly or indirectly as a source of energy. Thermoelectric or pyroelectric devices can instantly transform heat to electricity,^[^ ^204^ ^]^ while photothermal photocatalysis^[^ ^205^ ^]^ or piezo electronics can be used for the indirect transformation. Ho's group has directly and indirectly scavenged waste thermal energy for additional electricity generation during the evaporation process.^[^ ^206^ ^]^ The system relies upon a steam generation isolated structure (carbon sponge), whereas a ferroelectric fluoropolymer PVDF plating unit is employed for collecting water vapor based generated thermomechanical reaction interconnectivity of pyroelectric and piezoelectric effects. Measurements of temperature fluctuations corresponding to closed‐circuit current and open‐circuit voltage. This project offers a new opportunity to create safe or on‐site freshwater and electricity. Another research performed by Ho's Group^[^ ^36^ ^]^ is the coupled photothermally aided synthesis and hydrogen desalination by employing nano‐compounds of (SiO_2_/Ag@TiO_2_ core–shell) acting as thermal solar panels. Solar thermal nanocomposites are designed to overcome the significant challenges facing traditional catalytic systems by actively producing locally generating heat at the interface of reactive edges to reduce the heat and mass waste thermally. For outdoor research, photothermal enhanced real‐time catalysis and desalination under the real sun is validated by evidence of conception for hydrogen production and distillation process.

Furthermore, more energy generation systems caused by evaporation have been developed progressively and incorporated with solar evaporation system. Zhou et al. introduced to harvest simultaneous steam and power generation from seawater, used the evaporation‐driven salinity difference.^[^ ^176^ ^]^ Reformed filter paper with carbon nanotube which absorbs the solar energy and performs as ion‐selective membrane along with Nafion, assemble the hybrid system. The system demonstrated an effective steam production efficiency of up to 75% under one sun illumination 1 W m^−2^. They have revealed evaporative water flow in porous carbon materials for electricity generation. This technology can also be modified for water‐energy nexus with the efficient use of solar energy to generate water and energy simultaneously.^[^ ^176^, ^207^ ^]^ Ho's group demonstrated a hybrid system that exploits condensing processes in solar evaporation to generate energy. To simultaneously perform the freshwater production along with energy generation, a basic framework of triboelectric nanogenerator (TENG) has been developed.  The sealed system causes water vapor to condense on the wall, producing a triboelectric signal as the condensate flows down due to water electrification and with the polytetrafluoroethylene (PTFE) film adhered on the wall. Prototype bottom part is employed for storing condensate. Multidirectional mechanical energy could be collected by round bottom surface or tumble toy, such type of any motion/shake increases and causes the center of mass to move in various directions. This results in the production of triboelectricity because the condensate collected moves on the PTFE line surface. In such scenario, excess energy such as triboelectricity, simultaneously produced by two means (gravitational flow and condensate swinging) while the condensate is collected during solar evaporation. Increasing the productization of energy and water without further stressing the atmosphere is the definitive benefits of this relation. Also, multifunctional photo‐thermal materials can enable the use and conversion efficiency of solar power by hybrid‐compatible applications.^[^ ^208^ ^]^

## 6 Summary and Outlook

In this review paper, we have addressed the current development from the broad aspect and researcher's contribution towards nanoenabled photothermal absorbers based solar‐powered water evaporation system introducing new functional nanomaterials such as carbon‐based materials, plasmonic metallic nanoparticles (NPs), metal oxides, and conjugated polymers. A theoretical analysis of conduction, convection, and radiation along with salt rejection has been explored extensively for scheming nanoenabled photothermal materials utilizing minimum resources. For possessing unique properties latest nanoenabled photothermal absorbers that can revolutionize the former solar distillation method, making it a new green breakthrough for healthy drinking water production and extend their applications for upcoming challenges. Various approaches have been scrutinized to manufacture improved solar adsorption systems that enhance solar adsorption, control the variability of solar energy experiment conditions, considering highly efficient, low cost, and stable evaporation rate pursuing environmental issues. The invention of new nanoenabled photothermal materials providing new aspects such as longevity, eco‐friendly, robustness, thermal stability, and flexibility, etc. for future applications that are expected to grow in the coming years. On behalf of the brief analysis, few challenges are concluded for researchers in the selection of photothermal materials for clean water production.

1. During research into solar evaporation or distillation, no field‐approved guidelines were approved. An analysis of the literature shows that there is a notable difference in experimental and energy efficiency calculations, which makes it impossible for different photothermal materials only to be directly comparable with the literary values of solar energy efficiency and water evaporation rate.
2. The long‐term sustaining effectiveness, with photothermal material storage designs for tackling realistic water, such as actual seawater, groundwater, river water, and wastewater from manufacturing, of many photothermal materials and designs has not been verified. In seawater and hypersaline water, dissolved salts such as NaCl, CaSO_4_, and MgSO_4_ will crystallize, and their crystallization activity is unknown mainly during solar‐driven processes of solar evaporation and distillation. The impact of salinity fouling of Ca^2+^, Mg^2+,^ and other insoluble or slightly soluble materials in photothermal materials has not particularly been examined yet.  Most importantly, little consideration was taken to the expectation of biofouling photothermal materials resulting from organic dissolved materials and other bio‐species present in source waters.
3. Most water supply impaired sources consist of volatile organic compounds (VOCs), that evaporate during solar distillation and accumulate along with water. The VOC concentrations in the distillation collected can even be enhanced by distillation, the solar distillation heel of Achilles, and more advanced, multifunctional photothermal designs are needed.
4. A significant impact of uncertainty in solar light intensity on solar energy‐based evaporation and distillation has been ignored or insufficiently examined to date.
5. Since the current solar steam generation (SSG) is sufficiently high, the scope for further improvement is limited. Consequently, additional cost‐cutting is an essential factor for practical applications.  A big challenge remains to manufacture stable, recyclable, biodegradable, and durable nanomaterial photothermally for a low price. In the laboratory, the nanomaterial products are typically synthesized in limited amounts irrespective of the costs. This critical issue, though significant, has recently gained little attention. Yang et al. have attempted to expand the production of SSG photothermal paper to resolve this problem. Higher performance has been demonstrated with the highly flexible fire‐resistant HN/ CNT photothermal paper based on ultra‐long hydroxyapatite nanowire and carbon nanotubes (HN / CNT) in SSG additions. The scaled‐up production of the HN/CNT photothermal paper has been realized by employing the large‐scale synthesis of HNs using a large stainless‐steel autoclave with a volume of 100 L. The large‐scale nanomaterials showed stable recycling and long‐term usage performance inefficient production, simulation of harmful wastewater from real seawater, including industrial dyes, biological bacteria, and heavy metals.
6. Salty water, generally considered as marine water, is passed through an artificial treatment with SSG in laboratories. However, SSG facing real seawater consisting of many more complex components poses another challenge. Increased radiation time produces a significant amount of steam, while even the salt particles are crystallized, thus reducing the efficiency of evaporation by inhibiting further transport of water. So, practically SSG's applications are greatly influenced by the structural design of salt rejecting devices. Chen et al. demonstrated experimentally a solar energy‐based continual functioning evaporative system, capable of sailing over saline water while generating freshwater vapors. The evaporating system employed a white hydrophobic material with a porous structure to provide channels for water transportation to the top surface, whereas the hydrophobic membranes were also used to discharge and diffuse concentrated salt back into the water body.^[^ ^175^ ^]^ Except for salt rejecting structure, hydrophobic membranes were also used for rejecting salt. Zhao et al. suggested a hydrophobic membrane that includes a Ti_3_C_2_ nanosheet layer of silane modification for the harvest of sunlight (1*H*,1*H*,2*H*,2*H* perfluoro decyl) and a commercial water‐filling membrane of the container. SSG still has, however, significant challenges to sustainable desalination in the evaporation of water and waste disposal in complex seawater with high levels of efficiency. In this context, an evaporation rate of 1.31 kg m^−2^ h^−1^ was achieved along with steam conversion effectiveness of 71% under one sun illumination.

## Conflict of Interest

The authors declare no conflict of interest.
